# Exploring the Relationship Between Immune Cells and Chronic Kidney Disease by Mendelian Randomization, Colocalization Analysis, and SMR

**DOI:** 10.1155/mi/4279158

**Published:** 2025-04-16

**Authors:** Huiling Zhu, Chaofan Qin, Si Cheng, Xi Zhang

**Affiliations:** ^1^Department of Cardiology, The First Affiliated Hospital of Chongqing Medical University, Chongqing, China; ^2^Department of Orthopedics, The Second Affiliated Hospital of Chongqing Medical University, Chongqing, China; ^3^Department of Nephrology, The First Affiliated Hospital of Chongqing Medical University, Chongqing, China

**Keywords:** chronic kidney disease, colocalization analysis, immune cells, Mendelian randomization, SMR

## Abstract

**Background:** Chronic kidney disease (CKD) impacts millions of individuals annually. Current research suggests that immune factors played a significant role in CKD. However, the potential causal relationship between them remains unclear.

**Methods:** We conducted a comprehensive Mendelian randomization (MR) analysis to assess the potential causal association between 731 immune cells and CKD. Sensitivity analysis was performed to test for heterogeneity and horizontal pleiotropy, including the Cochran Q test, leave-one-out test, MR-Egger intercept analysis, and MR-PRESSO test. The bidirectional MR was utilized to investigate the bidirectional relationship between the immune cells and CKD. Multivariable MR was also conducted to mitigate confounding among immune cells. The colocalization analysis was performed to find the key genes of immune cells. We used the Summary data-based MR (SMR) analysis to generate effect estimates between the cis-eQTL and immune cells. The heterogeneity in dependent instruments (HEIDIs) test was used to test the heterogeneity between dependent instrumental variables.

**Results:** We identified 14 potential pathogenic factors and six potential protective factors through the univariable MR. Moreover, we did not find reverse causation by using the bidirectional MR. We finally identified one risk factor and two protective factors after multivariate MR adjustment for effects between immune cells. CD28 on CD28^+^ CD45RA^+^ CD8^+^ T cell could increase the risk of CKD (Pval: 0.033, OR: 1.112, 95% CI: 1.009–1.227). CD11c on myeloid dendritic cell (DC) could decrease the risk of CKD (Pval: 0.02, OR: 0.854, 95% CI: 0.748–0.975). CD45RA on naive CD4^+^ T cell could decrease the risk of CKD (Pval: 0.026, OR: 0.918, 95% CI: 0.852–0.990). Importantly, we observed no evidence of heterogeneity and pleiotropy, signifying the robustness of our results. *BACH2* (PPH4.abf = 0.999, P_SMR: <0.001, P_HIEDI: 0.132) and *HLA-G* (PPH4.abf = 0.990, P_SMR: <0.001, P_HIEDI: 0.141) shared the same variant with CD28 on CD28^+^ CD45RA^+^ CD8^+^ T cell. *PAQR9* (PPH4.abf = 0.992, P_SMR: <0.001, P_HIEDI: 0.215) shared the same variant with CD11c on myeloid DC.

**Conclusion:** MR identified a potential correlation between CKD and immune cells. Colocalization and SMR found the key genes of immune cells. Our findings offer insights into the prevention and management of CKD. However, further investigation is required to elucidate the precise mechanisms underlying this relationship.

## 1. Introduction

The glomerular filtration rate (GFR) of <60 mL/min/1.73 m^2^ is often considered a sign of chronic kidney disease (CKD) [1]. As the population ages, there will inevitably be a corresponding rise in the incidence of CKD and the associated demand for healthcare [2, 3]. CKD has a variety of causes, including diabetes, hypertension, infectious diseases and environmental toxins, and so on [4, 5]. However, its precise etiology remains unknown.

Research has indicated a significant role for the immune system in CKD [6]. Dendritic cells (DCs) are a type of antigen-presenting cell (APC). Research has shown that these cells could increase the activity of CD8^+^ T cells, which may have a role to play in promoting glomerular injury [7, 8]. The development of renal fibrosis and progressive CKD is associated with macrophages [9, 10]. Helper T cell 1 (Th1) cells have been found to undergo high levels of both OXOHOS and glycolytic metabolism, with a view to delaying renal fibrosis [11, 12]. However, Helper T cell 2 (Th2) cells could prompt renal fibrosis under diverse stimuli [11, 12]. The association between immune cells and CKD need to be further explored.

In epidemiology, Mendelian randomization (MR) is a method that utilizes measured genetic variation to confirm the causal effect of an exposure on an outcome [13, 14]. Genotype information is the instrumental variable of MR. MR employs Mendel's second law, and it can be conceptualized as a form of “natural randomized controlled trial.” MR is effective in reducing the effects of confounders and reverse link [13, 14]. Summary data-based MR (SMR) could test for pleiotropic association between the expression level of a gene and the complex trait by using summary-level data from genome-wide association studies (GWASs) and expression quantitative trait loci (eQTL) studies [15]. The SMR and heterogeneity in dependent instruments (HEIDIs) methodology can be interpreted as an analysis to test if the effect size of a single-nucleotide polymorphisms (SNPs) on the phenotype is mediated by gene expression [15]. The concept of colocalization analysis has been put forth as a means of identifying genetic drivers that are common across multiple omics [16]. In instances where the overlap of association signals in two or more omics is attributable to the sharing of a causal variant, a phenomenon known as colocalization positivity, the probability of the genetic variant being a causal variant is significantly enhanced [16].

In this study, the MR was conducted to investigate the relationship between immune cells and CKD. Meanwhile, we employed SMR and colocalization analyses to identify the key gene loci that regulate immune cells. The objective of this study is to identify the principal immune cells and genes that are involved in the development of CKD, in order to gain new insights into potential avenues for the treatment of CKD.

## 2. Materials and Methods

### 2.1. Study Design

**Figure 1:**
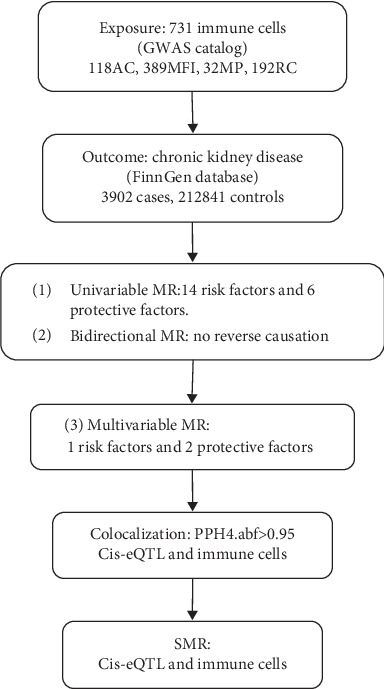
Schematic presentation of our study.

The analysis process is illustrated in Figure 1. Firstly, we conducted a univariable MR to explore the potential relationship between the immune cells and CKD [17, 18]. Secondly, to minimize the potential for reverse association, we performed bidirectional MR to explore the relationship between immune cells and CKD. Thirdly, recognizing that different immune cells may interact and influence each other due to genetic pleiotropy, potentially introducing confounding effects, we then performed multivariable MR analysis to assess the direct influence of immune cells on CKD [19, 20]. Among the positive cells screened by univariable MR, in order to further control for interactions between immune cells and to verify their independent causal effects, we conducted multivariable MR. Multivariable MR incorporates multiple positive immune cells into the model simultaneously to analyze the independent effects of each immune cell [17, 21]. Furthermore, we conducted the colocalization analysis and SMR to find the key gene for regulating the immune cells.

### 2.2. Data Source

The GWAS data for immune cells were acquired from the latest study involving 3757 individuals of Sardinian descent within the European population [22]. A total of 731 immunophenotypes were included in the study, comprising 118 absolute cell (AC) counts, 389 median fluorescence intensities (MFIs) reflecting surface antigen levels, 32 morphological parameters (MPs), and 192 relative cell (RC) counts [22]. The MFI, AC, and RC features encompass B cells, CDCs, mature stages of T cells, monocytes, myeloid cells, TBNK (T cells, B cells, and natural killer cells), and Treg panels, while the MP feature encompasses CDC and TBNK panels [22]. Twenty-two million genetic variations were measured in this research [22].

CKD data were obtained from the FinnGen database (https://www.finngen.fi/fi). This dataset encompasses 3902 cases, 212,841 controls, and 16,380,459 SNPs. CKD diagnoses were made according to the International Classification of Diseases, including ICD-10 (N18) and ICD-9 (585) coding standards. All of the population groups included in the database are of European origin. Other relevant information could be found on the FinnGen website (Risteys Home [finregistry.fi]).

The eQTLGen data set comprised 16,987 genes and 31,684 cis-eQTLs in blood samples from predominantly healthy European individuals. The eQTL data were obtained from the eQTLGen Consortium (https://eqtlgen.org/). A comprehensive account of the data can be found in the original article [23].

### 2.3. MR Analysis

#### 2.3.1. Selection of Instrumental Variables

We conducted various strict quality controls to select IVs that satisfy the three fundamental assumptions of MR analysis. This ensures the robustness and reliability of the MR analysis. Firstly, the SNPs of immune cells were selected at a genome-wide significance threshold (*p* < 5E-08) [13, 24]. The smaller the *p*-value, the stronger the association between the SNP and exposure, but there may be too few SNPs, resulting in insufficient statistical power. Conversely, the larger the *p*-value, the greater the likelihood of pleiotropy [24]. Secondly, we addressed the issue of linkage disequilibrium (LD) between SNPs by removing strongly linked variants (*r*^2^ = 0.001, clumping distance of 10,000 kb). LD, which means that alleles belonging to two or more genetic loci are more likely to occur on a chromosome at the same time than at random. This step aimed to mitigate any potential bias in the results caused by LD [13]. Lastly, we computed the F-statistics for all the selected SNPs. SNPs with F-statistics less than 10 were excluded to ensure that all remaining SNPs were strongly associated with the exposure [25]. F statistics were calculated using the formula *F* = beta^2^/standard error^2^(SE) [26, 27] (Supporting Information Table S1).

#### 2.3.2. Statistical Analysis

In our study, the inverse variance weighted (IVW) method was employed as our primary analytical method. The *p*-value less than 0.05 for IVW was deemed statistically significant. We conducted the MR-Egger test to assess the presence of the intercept because the IVW method assumes the absence of an intercept term [28]. Moreover, the MR-Egger, weighted median, weighted mode, and simple mode methods were conducted to enhance the robustness of our results.

We performed the Cochran's Q test and MR-Egger intercept analysis to ensure the absence of heterogeneity and pleiotropy. Heterogeneity was deemed present if the Q-*p*-value was less than 0.05 [29]. MR-Egger intercept *p*-value exceeding 0.05 indicated the absence of pleiotropy [30]. The MR-PRESSO test was employed with the objective of identifying and addressing pleiotropy, as well as removing outliers [31]. Eventually, the leave-one-out test was conducted to investigate whether the result was affected by a single SNP.

We conducted all statistical analyses using the “TwoSampleMR” (version 0.5.7) packages within the R statistical software (version 4.3.1).

### 2.4. Colocalisation Analysis

Colocalization analysis between cis-eQTLs and immune cell used the R package coloc [16]. These immune cells were confirmed by multivariable MR. Analyses were conducted using SNPs harmonized by the TwoSampleMR package with default prior probabilities: p1 = 1E−4, p2 = 1E−4, p12 = 1E−5. P1, p2, and p12 are predefined probabilities indicating the likelihood of a substantial link between the SNP in the test area and gene expression, immune cell function, or both.

The posterior probabilities derived from the colocalization analysis correspond to one of five hypotheses. (1) PPH0: SNPs are not associated with either trait. (2) PPH1: SNPs are associated with gene expression but not with the immune cell. (3) PPH2: SNPs are associated with the immune cell but not with gene expression. (4) PPH3: SNPs are associated with the immune cell and gene expression but are driven by different SNPs. (5) PPH4: SNPs are associated with the immune cell and gene expression was driven by common SNPs. The threshold for statistical significance in colocalization was set at PPH4 >0.95.

### 2.5. SMR Analysis

We performed SMR analysis to generate effect estimates [15]. Using summary-level data from GWAS and cis-eQTLs studies, we assessed the causal relationship between identified immune cells and gene expression levels. In addition, we used the SMR software package (V.1.03) to perform allele harmonization and analysis [15]. The *p*-value threshold for SMR analysis was 0.05.

The HEIDI method is used to detect heterogeneity between dependent instrumental variables, which can help differentiate between pleiotropy and linkage scenario [15]. The HEIDI test indicates that the association is caused by shared genetic variation if the *p*-value is greater than 0.05. The HEIDI test was also conducted by using the SMR software (V.1.03).

## 3. Result

### 3.1. Mendelian Randomization

#### 3.1.1. Univariable MR

In our study, we investigated the association between 731 immune cell and CKD (Supporting Information Table S2). Our analysis revealed that 47 of these immune cells were significantly associated with CKD (Supporting Information Table S3). However, we excluded 25 factors with fewer than three SNPs each due to limitations in the number of available SNPs (Supporting Information Table S3). HLA DR on CD14- CD16- was also excluded by us due to the existence of the pleiotropy (Supporting Information Table S4).

**Figure 2:**
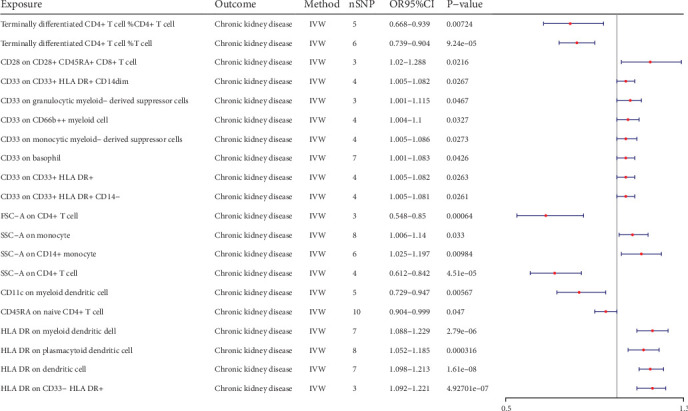
Forest plots of effects of immune cells on the CKD by using univariable MR.

Consequently, we identified 14 potential pathogenic factors and six potential protective factors (Figure 2, Table 1). Terminally differentiated CD4^+^ T cell %CD4^+^ T cell could decrease the risk of CKD (Pval: 0.007, OR: 0.792, 95% CI: 0.668–0.939). Terminally differentiated CD4^+^ T cell %T cell could decrease the risk of CKD (Pval: <0.001, OR: 0.818, 95% CI: 0.739–0.904). CD28 on CD28^+^ CD45RA^+^ CD8^+^ T cell could increase the risk of CKD (Pval: 0.022, OR: 1.146, 95% CI: 1.02–1.288). CD33 on CD33^+^ HLA DR^+^ CD14^dim^ could increase the risk of CKD (Pval: 0.027, OR: 1.043, 95% CI: 1.005–1.082). CD33 on granulocytic myeloid-derived suppressor cells could increase the risk of CKD (Pval: 0.047, OR: 1.056, 95% CI: 1.001–1.115). CD33 on CD66b^++^ myeloid cell could increase the risk of CKD (Pval: 0.033, OR: 1.051, 95% CI: 1.004–1.1). CD33 on monocytic myeloid-derived suppressor cells could increase the risk of CKD (Pval: 0.027, OR: 1.045, 95% CI: 1.005–1.086). CD33 on basophil could increase the risk of CKD (Pval: 0.043, OR: 1.041, 95% CI: 1.001–1.083). CD33 on CD33^+^ HLA DR^+^ could increase the risk of CKD (Pval: 0.026, OR: 1.043, 95% CI: 1.005–1.082). CD33 on CD33^+^ HLA DR^+^ CD14^−^ could increase the risk of CKD (Pval: 0.026, OR: 1.042, 95% CI: 1.005–1.081). FSC-A on CD4^+^ T cell could decrease the risk of CKD (Pval: <0.001, OR: 0.682, 95% CI: 0.548–0.85). SSC-A on monocyte could increase the risk of CKD (Pval: 0.033, OR: 1.071, 95% CI: 1.006–1.14). SSC-A on CD14^+^ monocyte could increase the risk of CKD (Pval: 0.01, OR: 1.108, 95% CI: 1.025–1.197). SSC-A on CD4^+^ T cell could decrease the risk of CKD (Pval: <0.001, OR: 0.718, 95% CI: 0.612–0.842). CD11c on myeloid DC could decrease the risk of CKD (Pval: 0.006, OR: 0.831, 95% CI: 0.729–0.947). CD45RA on naive CD4^+^ T cell could decrease the risk of CKD (Pval: 0.047, OR: 0.95, 95% CI: 0.904–0.999). HLA DR on myeloid DC could increase the risk of CKD (Pval: <0.001, OR: 1.157, 95% CI: 1.088–1.229). HLA DR on plasmacytoid DC could increase the risk of CKD (Pval: <0.001, OR: 1.116, 95% CI: 1.052–1.185). HLA DR on DC could increase the risk of CKD (Pval: <0.001, OR: 1.154, 95% CI: 1.098–1.213). HLA DR on CD33^−^ HLA DR^+^ could increase the risk of CKD (Pval: <0.001, OR: 1.155, 95% CI: 1.092–1.221).

#### 3.1.2. Bidirectional MR

We performed bidirectional MR to rule out reverse causality between immune cells and CKD. There was no bidirectional relationship between the immune cells and CKD (Supporting Information Table S5).

#### 3.1.3. Multivariable MR

**Figure 3:**
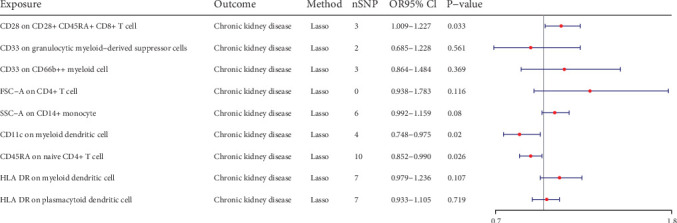
Forest plots of effects of immune cells on the CKD by using multivariable MR.

We conducted the multivariable MR to investigate the independent effect size of immune cells on CKD (Figure 3). The 20 immune cells included in multivariate MR were identified by univariate MR. We finally identified one risk factor and two protective factors after multivariate MR adjustment for effects between immune cells. CD28 on CD28^+^ CD45RA^+^ CD8^+^ T cell could increase the risk of CKD (Pval: 0.033, OR: 1.112, 95% CI: 1.009–1.227). CD11c on myeloid DC could decrease the risk of CKD (Pval: 0.02, OR: 0.854, 95% CI: 0.748–0.975). CD45RA on naive CD4^+^ T cell could decrease the risk of CKD (Pval: 0.026, OR: 0.918, 95% CI: 0.852–0.990).

#### 3.1.4. Sensitivity Analysis

**Figure 4:**
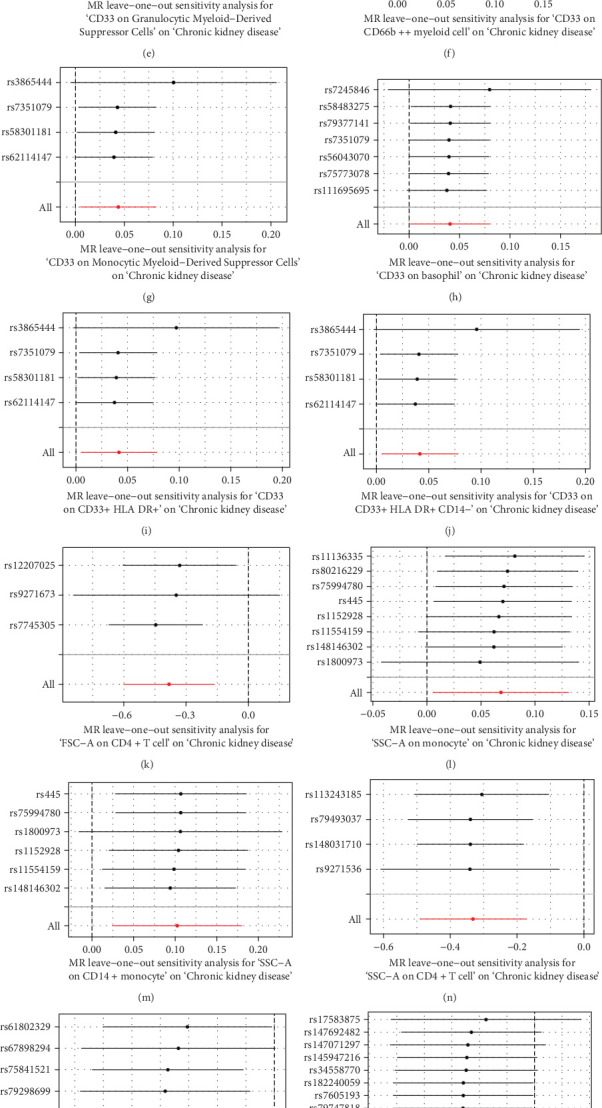
The leave one out plot of univariable MR. (a) Terminally differentiated CD4^+^ T cell %CD4^+^ T cell; (b) terminally differentiated CD4^+^ T cell %T cell; (c) CD28 on CD28^+^ CD45RA^+^ CD8^+^ T cell; (d) CD33 on CD33^+^ HLA DR^+^ CD14^dim^; (e) CD33 on granulocytic myeloid-derived suppressor cells; (f) CD33 on CD66b^++^ myeloid cell; (g) CD33 on monocytic myeloid-derived suppressor cells; (h) CD33 on basophil; (i) CD33 on CD33^+^ HLA DR^+^; (j) CD33 on CD33^+^ HLA DR^+^ CD14^−^; (k) FSC-A on CD4^+^ T cell; (l) SSC-A on monocyte; (m) SSC-A on CD14^+^ monocyte; (n) SSC-A on CD4^+^ T cell; (o) CD11c on myeloid dendritic cell; (p) CD45RA on naive CD4^+^ T cell; (q) HLA DR on myeloid dendritic cell; (r) HLA DR on plasmacytoid dendritic cell; (s) HLA DR on dendritic cell; (t): HLA DR on CD33- HLA DR^+^.

The Cochrane's Q test and the MR-Egger intercept test were employed in the MR analysis. The *p*-value of the Cochrane's Q test exceeded 0.05, indicating that our results did not have heterogeneity (Table 2). The *p*-value of the MR-Egger intercept test exceeded 0.05, demonstrated that our results did not have horizontal pleiotropy (Table 2). MR-PRESSO analysis showed no outliers or pleiotropy (Table 2). However, the MR-PRESSO analysis was not performed for CD28 on CD28^+^ CD45RA^+^ CD8^+^ T cell, CD33 on granulocytic myeloid-derived suppressor cells, FSC-A on CD4^+^ T cell and HLA DR on CD33^−^ HLA DR^+^ due to the limited number of SNPs. Moreover, the leave-one-out test indicated that the results did not affect by individual SNP (Figure 4).

### 3.2. Colocalization Analysis

We explored the shared variant between the immune cells and cis-eQTL by employing the colocalization analysis. Colocalization analysis strongly suggested that *BACH2* (PPH4.abf = 0.999), *TRIM26* (PPH4.abf = 0.991) and *HLA-G* (PPH4.abf = 0.990) shared the same variant with CD28 on CD28^+^ CD45RA^+^ CD8^+^ T cell (Table 3). *PAQR9* (PPH4.abf = 0.992) and *RASA3* (PPH4.abf = 0.992) shared the same variant with CD11c on myeloid DC (Table 3). *ZBTB12* (PPH4.abf = 0.976) and *FOXP1* (PPH4.abf = 0.961) shared the same variant with CD45RA on naive CD4^+^ T cell (Table 3).

### 3.3. Summary Data-Based MR Analysis

We performed the SMR analysis by utilizing the blood cis-eQTLs data to validate the result of colocalization analysis. *BACH2* (P_SMR: <0.001, P_HIEDI: 0.132), *TRIM26* (P_SMR: <0.001, P_HIEDI: 0.007), and *HLA-G* (P_SMR: <0.001, P_HIEDI: 0.141) were associated with CD28 on CD28^+^ CD45RA^+^ CD8^+^ T cell. *PAQR9* (P_SMR: <0.001, P_HIEDI: 0.215) and *RASA3* (P_SMR: <0.001, P_HIEDI: 0.027) were associated with CD11c on myeloid DC. *ZBTB12* and *FOXP1*were not associated with CD45RA on naive CD4^+^ T cell by utilizing the SMR analysis. The HIEDI test indicated that the association between *TRIM26* and *RASA3* and immune cells was caused by LD (P_HIEDI: <0.05).

## 4. Discussion

In this study, we investigated the potential relationship between the immune cells and CKD by utilizing the univariable and multivariable MR. We found 14 risk factors and six protective factors through the univariable MR. However, only three immune cells remained associated with CKD after eliminating the interaction between immune cells, including one risk factor and two protective factors. *BACH2* and *HLA-G* shared the same variant with CD28 on CD28^+^ CD45RA^+^ CD8^+^ T cell which could increase the risk of scoliosis. *PAQR9* shared the same variant with CD11c on myeloid DC which could decrease the risk of scoliosis. CD45RA on naive CD4^+^ T cell could decrease the risk of scoliosis.

In this study, we found that CD28 on CD28^+^ CD45RA^+^ CD8^+^ T cell could accelerate the development of CKD. CD28 is a member of a subfamily of costimulatory molecules, which is characterized by the presence of extracellular variable immunoglobulin-like structural domains [32]. CD28 is expressed on ~50% of CD8^+^ T cells. In humans, the proportion of CD28^−^-positive T cells declines with age [32]. In renal transplant recipients, CD8^+^ CD28^−^ T cells may serve a protective function against organ rejection, suggesting a potential immunosuppressive role for this subset [33, 34]. CD8^+^ T cells activate macrophages by secreting IFN-*γ* and TNF-*α*. Activated macrophages are able to secrete pro-inflammatory factors, which in turn leads to enhanced inflammation in the kidney, resulting in kidney injury [35]. Therefore, we think CD8^+^ CD28^+^ T cells may play a risk role in CKD.

The evidence indicates that *BACH2* plays a role in regulating the terminal differentiation of CD8^+^ T cells [36, 37]. This is achieved by limiting the access of AP-1 transcription factors to enhancers, which in turn enhances the immune response capacity of T cells [37]. The deletion of *BACH2* has been shown to result in the over-differentiation and functional enhancement of CD8^+^ T cells, which further supports its importance in regulating immune responses [37]. *BACH2* is also implicated in the regulation of regulatory T cell (Treg)-mediated immune homeostasis [38]. Mice lacking BACH2 exhibit lethal lung and intestinal inflammation. *BACH2* prevents lethal autoimmunity through its role in Treg cell formation. However, *BACH2* has been demonstrated to suppress the C–C motif chemokine receptor 4 (CCR4), growth stimulation-expressed gene 2 (ST-2), S100 calcium-binding protein a (S100a), and Blimp-1, thereby maintaining the naive T-cell state and consequently suppressing the body's immune status [36, 39]. Further investigation is required to elucidate the mechanism of action of *BACH2*.

*HLA-G* was initially conceptualized as a conventional immune-regulatory molecule that suppresses the cytotoxic activity of maternal immune cells towards the fetal [40]. HLA-G (pos) T cells are found in inflamed skeletal muscle in myositis and the cerebrospinal fluid in people with acute inflammatory disorders. This suggests that they play an important role in controlling inflammation in the body [41]. We speculate that in the kidney, the immunosuppressive effects of HLA-G may be impaired, leading to the promotion of CKD.

Our study demonstrated that CD45RA on naive CD4^+^ T cell monocytes was associated with a reduced risk of CKD. Human CD45 mRNA has six isoforms and naive T cell is well known to express CD45RA [42, 43]. CD45 has been demonstrated to inhibit the activity of Src family kinase (SFK) by dephosphorylating their activation sites, thereby negatively regulating immune cell activation [44, 45]. It is postulated that CD45 may be capable of mitigating renal impairment by inhibiting the function of immune cells. However, it should be noted that CD45 is also capable of activating the body's immune system and thereby initiating the immune response [42, 44]. Therefore, its specific mechanisms need to be further explored.

We also revealed that CD11c on myeloid DC could delay the development of A CKD. CD11c has been identified as a DC marker for several decades [46]. CD11c DCs can mediate peripheral immunity suppression induced by renal ischemia–reperfusion injury [47]. Patients with CKD stage III had significantly lower DC cells than the normal group [8]. DC cells in renal lymph nodes are able to present self-antigens to CTL cells, thereby inducing immune tolerance and reducing the attack of immune cells on the kidney [48, 49]. The role of *PAQR9* in immune cells remains to be elucidated, and further research is necessary to ascertain the mechanisms involved.

There were several strengths in our study. Primarily, multiple approaches were conducted in our analysis, including univariable, bidirectional, multivariable MR and the SMR analysis, and colocalization analysis. Secondly, we used multiple sensitivity analyses to increase the credibility of the results. Thirdly, the study was conducted exclusively on a European population to minimize the impact of ethnicity.

There were also some limitations in this study. It is important to note that the conclusion may only be applicable to European populations. Differences in ethnicity may lead to biased results, so further research is needed before it can be applied to other ethnic groups. Additionally, the sample size of the data selected for analysis was limited. Finally, the Bonferroni correction was not utilized in this research. Our aim was to find goals related to the prevention and treatment of CKD. However, the Bonferroni criteria are too strict and may cause us to miss meaningful results. Although this could increase the likelihood of the type I error, we conducted the multivariate MR, which could control for some of the false positive rate. However, there are still some unobserved confounders that can affect our results.

## 5. Conclusion

We have utilized MR, colocalization, and SMR to find the immune cells associated with CKD and have found genes strongly associated with the immune cells. It provides a new therapeutic idea for the immunotherapy of CKD. Nevertheless, the relevant mechanism is still unclear. The result of our study could provide a foundation for future research.

## Figures and Tables

**Table 1:** Univariable MR immune cells on chronic kidney disease.

Exposure	Outcome	b	pval	or	or_lci95	or_uci95
Terminally differentiated CD4^+^ T cell %CD4^+^ T cell	Chronic kidney disease	−0.233	7.24E-03	0.792	0.668	0.939
Terminally differentiated CD4^+^ T cell %T cell	Chronic kidney disease	−0.201	9.24E-05	0.818	0.739	0.904
CD28 on CD28^+^ CD45RA^+^ CD8^+^ T cell	Chronic kidney disease	0.137	2.16E-02	1.146	1.020	1.288
CD33 on CD33^+^ HLA DR^+^ CD14dim	Chronic kidney disease	0.042	2.67E-02	1.043	1.005	1.082
CD33 on granulocytic myeloid-derived suppressor cells	Chronic kidney disease	0.055	4.67E-02	1.056	1.001	1.115
CD33 on CD66b^++^ myeloid cell	Chronic kidney disease	0.050	3.27E-02	1.051	1.004	1.100
CD33 on monocytic myeloid-derived suppressor cells	Chronic kidney disease	0.044	2.73E-02	1.045	1.005	1.086
CD33 on basophil	Chronic kidney disease	0.041	4.26E-02	1.041	1.001	1.083
CD33 on CD33^+^ HLA DR^+^	Chronic kidney disease	0.042	2.63E-02	1.043	1.005	1.082
CD33 on CD33^+^ HLA DR^+^ CD14^−^	Chronic kidney disease	0.042	2.61E-02	1.042	1.005	1.081
FSC-A on CD4^+^ T cell	Chronic kidney disease	−0.382	6.40E-04	0.682	0.548	0.850
SSC-A on monocyte	Chronic kidney disease	0.068	3.30E-02	1.071	1.006	1.140
SSC-A on CD14^+^ monocyte	Chronic kidney disease	0.102	9.84E-03	1.108	1.025	1.197
SSC-A on CD4^+^ T cell	Chronic kidney disease	−0.332	4.51E-05	0.718	0.612	0.842
CD11c on myeloid dendritic cell	Chronic kidney disease	−0.185	5.67E-03	0.831	0.729	0.947
CD45RA on naive CD4^+^ T cell	Chronic kidney disease	−0.051	4.70E-02	0.950	0.904	0.999
HLA DR on myeloid dendritic cell	Chronic kidney disease	0.145	2.79E-06	1.157	1.088	1.229
HLA DR on plasmacytoid dendritic cell	Chronic kidney disease	0.110	3.16E-04	1.116	1.052	1.185
HLA DR on dendritic cell	Chronic kidney disease	0.143	1.61E-08	1.154	1.098	1.213
HLA DR on CD33^−^ HLA DR^+^	Chronic kidney disease	0.144	4.93E-07	1.155	1.092	1.221

**Table 2:** The sensitivity analyses of the univariable MR.

Exposure	MR-Egger intercept test		Cochran's Q test		MR-PRESSO	
	Egger_intercept	pval	Q	Q_pval	Outlier	pval
Terminally differentiated CD4^+^ T cell %CD4^+^ T cell	−0.109	0.119	7.248	0.123	0	0.225
Terminally differentiated CD4^+^ T cell %T cell	−0.06	0.124	6.168	0.29	0	0.364
CD28 on CD28^+^ CD45RA^+^ CD8^+^ T cell	−0.111	0.603	0.598	0.742	NA	NA
CD33 on CD33^+^ HLA DR^+^ CD14dim	0.03	0.357	1.569	0.666	0	0.497
CD33 on granulocytic myeloid-derived suppressor cells	0.028	0.513	1.032	0.597	NA	NA
CD33 on CD66b^++^ myeloid cell	0.016	0.562	0.952	0.813	0	0.72
CD33 on monocytic myeloid-derived suppressor cells	0.03	0.353	1.61	0.657	0	0.545
CD33 on basophil	0.023	0.558	1.69	0.946	0	0.771
CD33 on CD33^+^ HLA DR^+^	0.03	0.36	1.542	0.673	0	0.541
CD33 on CD33^+^ HLA DR^+^ CD14^−^	0.03	0.362	1.53	0.675	0	0.532
FSC-A on CD4^+^ T cell	0.218	0.538	2.26	0.323	NA	NA
SSC-A on monocyte	−0.04	0.155	6.188	0.518	0	0.603
SSC-A on CD14^+^ monocyte	−0.011	0.779	2.431	0.787	0	0.88
SSC-A on CD4^+^ T cell	−0.157	0.761	2.456	0.483	0	0.699
CD11c on myeloid dendritic cell	−0.059	0.356	2.236	0.692	0	0.756
CD45RA on naive CD4^+^ T cell	0.044	0.148	7.295	0.606	0	0.661
HLA DR on myeloid dendritic cell	−0.056	0.115	7.118	0.31	0	0.346
HLA DR on plasmacytoid dendritic cell	−0.021	0.472	12.848	0.076	0	0.244
HLA DR on dendritic cell	−0.014	0.594	5.541	0.476	0	0.576
HLA DR on CD33^−^ HLA DR^+^	−0.039	0.486	1.288	0.525	NA	NA

Abbreviation: NA, not available.

**Table 3:** The result of the colocalization analysis and SMR between cis-eQTL and immune cells.

cis-eQTL	Immune cells	Colocalization analysis	SMR	
		PPH4.abf	P_SMR	P_HEIDI
BACH2	CD28 on CD28^+^ CD45RA^+^ CD8^+^ T cell	0.999	1.13e-41	1.32e-01
TRIM26		0.991	4.75e-05	6.63e-03
HLA-G		0.990	2.35e-05	1.41e-01
PAQR9	CD11c on myeloid Dendritic cell	0.992	1.09e-04	2.51e-01
RASA3		0.992	1.86e-05	2.70e-02
ZBTB12	CD45RA on naive CD4^+^ T cell	0.976	NA	NA
FOXP1		0.951	5.70e-02	4.049e-01

Abbreviation: NA, not available.

## Data Availability

The data that support the findings of this study are openly available in GWAS Catalog (https://www.ebi.ac.uk/gwas), IEU Open GWAS (https://gwas.mrcieu.ac.uk), eQTLGen Consortium (https://www.eqtlgen.org/cis-eqtls.html), and FinnGen database (https://www.finngen.fi/en).

## Supporting Information

Supporting Information 1 Supporting Information Table S1: Information on univariable MR SNPs.

Supporting Information 2 Supporting Information Table S2: The results of univariable MR between the 731 immune cells and CKD.

Supporting Information 3 Supporting Information Table S3: The significant results of univariable MR between the 47 immune cells and CKD.

Supporting Information 4 Supporting Information Table S4: The MR result, the Cochran's Q test and the MR-Egger intercept test of HLA DR on CD14- CD16-.

Supporting Information 5 Supporting Information Table S5: The results of bidirectional MR between CKD and immune cells.
